# Supplementary material for: 5-hydroxymethylcytosine is highly dynamic across human fetal brain development
Source: BMC Genomics. 2017 Sep 18;18:738. doi: 10.1186/s12864-017-4091-x (PMC5604137; doi:10.1186/s12864-017-4091-x)
Supplement: Supplementary file 1 — Supplementary Tables. (ZIP 82808 kb) [file 12864_2017_4091_MOESM1_ESM.zip › 3. Supplementary Tables.docx]

Table S1: Number of probes passing our 5hmC detection threshold (Δβ_BS-oxBS_ > 0.036) as a function of the number of samples. See also Fig. 1a.

| **N samples** | **N probes**  **(All)** | **N probes**  **(Autosomal)** |  | **N samples** | **N probes**  **(All)** | **N probes**  **(Autosomal)** |
| --- | --- | --- | --- | --- | --- | --- |
| **0** | 103501 | 103063 |  | **36** | 3205 | 3134 |
| **1** | 22032 | 21751 |  | **37** | 3252 | 3173 |
| **2** | 10875 | 10574 |  | **38** | 3244 | 3182 |
| **3** | 7070 | 6664 |  | **39** | 3251 | 3176 |
| **4** | 5695 | 5280 |  | **40** | 3364 | 3295 |
| **5** | 4870 | 4426 |  | **41** | 3370 | 3297 |
| **6** | 4498 | 4022 |  | **42** | 3334 | 3246 |
| **7** | 4327 | 3851 |  | **43** | 3308 | 3242 |
| **8** | 4242 | 3816 |  | **44** | 3315 | 3236 |
| **9** | 4179 | 3794 |  | **45** | 3286 | 3213 |
| **10** | 4196 | 3850 |  | **46** | 3343 | 3275 |
| **11** | 4226 | 3935 |  | **47** | 3575 | 3508 |
| **12** | 4105 | 3895 |  | **48** | 3422 | 3359 |
| **13** | 4193 | 3987 |  | **49** | 3509 | 3437 |
| **14** | 4132 | 3971 |  | **50** | 3561 | 3481 |
| **15** | 4061 | 3929 |  | **51** | 3577 | 3497 |
| **16** | 4155 | 3996 |  | **52** | 3651 | 3577 |
| **17** | 4019 | 3894 |  | **53** | 3757 | 3684 |
| **18** | 4049 | 3966 |  | **54** | 3705 | 3634 |
| **19** | 3816 | 3704 |  | **55** | 3937 | 3873 |
| **20** | 3860 | 3774 |  | **56** | 3939 | 3865 |
| **21** | 3710 | 3623 |  | **57** | 4068 | 4008 |
| **22** | 3729 | 3637 |  | **58** | 4085 | 4018 |
| **23** | 3721 | 3645 |  | **59** | 4201 | 4140 |
| **24** | 3551 | 3464 |  | **60** | 4394 | 4335 |
| **25** | 3547 | 3472 |  | **61** | 4387 | 4325 |
| **26** | 3433 | 3369 |  | **62** | 4528 | 4472 |
| **27** | 3412 | 3336 |  | **63** | 4676 | 4626 |
| **28** | 3456 | 3373 |  | **64** | 4720 | 4679 |
| **29** | 3465 | 3396 |  | **65** | 4850 | 4802 |
| **30** | 3413 | 3337 |  | **66** | 4878 | 4834 |
| **31** | 3445 | 3381 |  | **67** | 5113 | 5085 |
| **32** | 3372 | 3293 |  | **68** | 5132 | 5103 |
| **33** | 3274 | 3198 |  | **69** | 5011 | 4989 |
| **34** | 3318 | 3246 |  | **70** | 5051 | 5029 |
| **35** | 3306 | 3233 |  | **71** | 5073 | 5061 |
|  |  |  |  | **Total:** | 411325 | 402035 |

|  | Total probes | Probes not passing detection threshold in all samples (%) | Enrichment  (95% CI) | *P*-value | Probes passing detection threshold in >1 sample (%) | Enrichment  (95% CI) | *P*-value | Probes passing detection threshold in all samples (%) | Enrichment  (95% CI) | *P*-value |
| --- | --- | --- | --- | --- | --- | --- | --- | --- | --- | --- |
| All autosomal probes | 402,035 | 103,063 (25.64) | - | - | 298,972 (74.36) | - | - | 5061 (1.26) | - | - |
| CpG island feature |  |  |  |  |  |  |  |  |  |  |
| Island | 132,168 | 76,301 (57.73) | 3.96 (3.91 - 4.01) | <1.00E-200 | 55,867 (42.27) | 0.25 (0.25 - 0.26) | <1.00E-200 | 87 (0.07) | 0.05 (0.04 - 0.06) | <1.00E-200 |
| Shore | 94,855 | 19,521 (20.58) | 0.75 (0.74 - 0.76) | 9.80E-239 | 75,334 (79.42) | 1.33 (1.31 - 1.35) | 9.80E-239 | 1658 (1.75) | 1.40 (1.32 - 1.48) | 7.02E-30 |
| Shelf | 36,510 | 957 (2.62) | 0.08 (0.07 - 0.08) | <1.00E-200 | 35,553 (97.38) | 12.81 (12.00 - 13.67) | <1.00E-200 | 654 (1.79) | 1.43 (1.32 - 1.55) | 2.64E-16 |
| Not island/shore/shelf | 138,502 | 6284 (4.54) | 0.14 (0.13 - 0.14) | <1.00E-200 | 132,218 (95.46) | 7.25 (7.06 - 7.45) | <1.00E-200 | 2662 (1.92) | 1.54 (1.47 - 1.61) | 1.41E-67 |
| Gene feature |  |  |  |  |  |  |  |  |  |  |
| TSS1500 | 71,792 | 26,305 (36.64%) | 1.68 (1.65 - 1.71) | <1.00E-200 | 45,487 (63.36) | 0.60 (0.59 - 0.61) | <1.00E-200 | 1057 (1.47) | 1.17 (1.10 - 1.25) | 4.69E-06 |
| TSS200 | 55,289 | 33,444 (60.49%) | 4.44 (4.36 - 4.52) | <1.00E-200 | 21,845 (39.51) | 0.23 (0.22 - 0.23) | <1.00E-200 | 128 (0.23) | 0.18 (0.15 - 0.22) | 6.64E-143 |
| 5' UTR | 56,764 | 25,265 (44.51%) | 2.33 (2.29 - 2.37) | <1.00E-200 | 31,499 (55.49) | 0.43 (0.42 - 0.44) | <1.00E-200 | 672 (1.18) | 0.94 (0.87 - 1.02) | 1.35E-01 |
| 1st Exon | 34,208 | 20,272 (59.26%) | 4.22 (4.12 - 4.32) | <1.00E-200 | 13,936 (40.74) | 0.24 (0.23 - 0.24) | <1.00E-200 | 93 (0.27) | 0.21 (0.17 - 0.26) | 2.04E-82 |
| Gene body | 147,417 | 21,257 (14.42%) | 0.49 (0.48 - 0.50) | <1.00E-200 | 126,160 (85.58) | 2.05 (2.01 - 2.08) | <1.00E-200 | 2262 (1.53) | 1.22 (1.16 - 1.29) | 7.69E-15 |
| 3' UTR | 16,431 | 834 (5.08%) | 0.16 (0.14 - 0.17) | <1.00E-200 | 15,597 (94.92) | 6.45 (6.01 - 6.92) | <1.00E-200 | 198 (1.21) | 0.96 (0.83 - 1.10) | 5.68E-01 |

Table S2: Differing CpG island and genic density of probes displaying detectable DNA hydroxymethylation in none vs. all samples

Table S3: Mean DNA hydroxymethylation (%) across autosomal sites by genomic feature. See also Fig. S1. *P*-value is provided for Mann-Whitney-Wilcoxon test (comparing probes within genomic feature to all probes).

|  |  | | **All samples**  **(n = 71)** | |  | |
| --- | --- | --- | --- | --- | --- | --- |
|  | **Total probes** | | **Mean 5hmC (%) (SD)** | | ***P*-value** | |
| **All probes** | 298,972 | | 4.16 (5.43) | | - | |
| **CpG island feature** | |  | |  | |  |
| Island | 55,867 | | 1.56 (3.97) | | <1.00E-200 | |
| Shore | 75,334 | | 3.91 (5.57) | | <1.00E-200 | |
| Shelf | 35,553 | | 4.91 (5.32) | | <1.00E-200 | |
| Not island/shore/shelf | 132,218 | | 5.20 (5.53) | | <1.00E-200 | |
| **Gene feature** | |  | |  | |  |
| TSS1500 | 45,487 | | 3.93 (5.53) | | <1.00E-200 | |
| TSS200 | 21,845 | | 2.14 (4.52) | | <1.00E-200 | |
| 5' UTR | 31,499 | | 3.85 (5.50) | | <1.00E-200 | |
| 1st Exon | 13,936 | | 2.24 (4.50) | | <1.00E-200 | |
| Gene body | 126,160 | | 4.37 (5.47) | | <1.00E-200 | |
| 3' UTR | 15,597 | | 4.74 (5.27) | | <1.00E-200 | |

|  | **Total probes** | **Significant**  **(*P* < 5e-05) probes (%)** | **Enrichment**  **(95% CI)** | ***P*-value** | **Hyper-hydroxymethylated (%)** | **Hypo-**  **hydroxymethylated probes (%)** | **Enrichment hyperhydroxymethylated**  **(95% CI)** | ***P*-value** |
| --- | --- | --- | --- | --- | --- | --- | --- | --- |
| **All probes** | 298,972 | 2181 (0.73) |  | - | 903 (41.40) | 1278 (58.60) | 0.71 (0.63 – 0.80) | 1.29E-08 |
| **CpG island feature** | |  |  |  |  |  |  |  |
| Island | 55,867 | 176 (0.32) | 0.43 (0.37 - 0.50) | 1.43E-33 | 102 (57.95) | 74 (42.05) | 1.38 (0.89 - 2.15) | 1.64E-01 |
| Shore | 75,334 | 531 (0.70) | 0.97 (0.88 - 1.06) | 4.86E-01 | 206 (38.79) | 325 (61.21) | 0.63 (0.49 - 0.81) | 2.65E-04 |
| Shelf | 35,553 | 261 (0.73) | 1.01 (0.88 - 1.15) | 9.21E-01 | 116 (44.44) | 145 (55.56) | 0.80 (0.56 - 1.15) | 2.20E-01 |
| Not island/shore/shelf | 132,218 | 1213 (0.92) | 1.26 (1.17 - 1.35) | 2.27E-10 | 479 (39.49) | 734 (60.51) | 0.65 (0.55 - 0.77) | 2.10E-07 |
|  |  |  |  |  |  |  |  |  |
| **Gene feature** | |  |  |  |  |  |  |  |
| TSS1500 | 45,487 | 267 (0.59) | 0.80 (0.70 - 0.91) | 6.32E-04 | 125 (46.82) | 142 (53.18) | 0.88 (0.62 - 1.25) | 4.89E-01 |
| TSS200 | 21,845 | 104 (0.48) | 0.65 (0.53 - 0.79) | 6.50E-06 | 41 (39.42) | 63 (60.58) | 0.65 (0.36 - 1.17) | 1.63E-01 |
| 5' UTR | 31,499 | 230 (0.73) | 1.00 (0.87 - 1.15) | 9.72E-01 | 74 (32.17) | 156 (67.83) | 0.48 (0.32 - 0.70) | 1.44E-04 |
| 1st Exon | 13,936 | 73 (0.52) | 0.72 (0.56 - 0.91) | 4.06E-03 | 30 (41.10) | 43 (58.90) | 0.70 (0.34 - 1.42) | 3.19E-01 |
| Gene body | 126,160 | 948 (0.75) | 1.03 (0.95 - 1.11) | 4.44E-01 | 391 (41.24) | 557 (58.76) | 0.70 (0.58 - 0.85) | 1.55E-04 |
| 3' UTR | 15,597 | 96 (0.62) | 0.84 (0.68 - 1.03) | 1.10E-01 | 46 (47.92) | 50 (52.08) | 0.92 (0.50 - 1.68) | 8.85E-01 |
|  |  |  |  |  |  |  |  |  |
| DNase 1 hypersensitivity sites | 111,494 | 1072 (0.96) | 1.32 (1.23 – 1.42) | 2.62E-13 | 459 (42.82) | 613 (57.18) | 0.75 (0.63 - 0.89) | 9.93E-04 |
| Transcription factor binding sites | 106,319 | 842 (0.79) | 1.09 (1.00 – 1.18) | 4.41E-02 | 354 (42.09) | 488 (58.03) | 0.73 (0.60 - 0.88) | 1.25E-03 |
| Alternative transcription events | 105,367 | 759 (0.72) | 0.99 (0.91 – 1.07) | 7.84E-01 | 329 (43.35) | 430 (56.65) | 0.77 (0.62 - 0.94) | 1.01E-02 |

Table S6: The distribution and direction of effect of dDHPs is not equal across genomic regions. See also Fig. S6.

Table S7: Summary of differentially hydroxymethylated regions (DHRs) associated with fetal brain development and sex, by chromosome. Spatially-correlated regions (Sidak corrected *P* < 0.05, number of probes ≥ 3) of differential DNA hydroxymethylation were identified with *comb-p* (using the parameters: seed = E-4; distance = 500). See Table S8 for a full list of dDHRs and Table S14 for a full list of sex DHRs.

|  | **Brain development**  **(dDHRS)** | **Sex**  **(sDHRs)** |
| --- | --- | --- |
| **Regions (n)** | 254 | 144 |
| **Mean probes per region (n)** | 5.43 | 5.49 |
| **Mean region size (bp) (SD)** | 551.30 (362.97) | 576.38 (311.86) |
| **Number of regions by chromosome:** |  |  |
| **1** | 25 | 0 |
| **2** | 17 | 0 |
| **3** | 6 | 0 |
| **4** | 8 | 0 |
| **5** | 12 | 0 |
| **6** | 24 | 0 |
| **7** | 32 | 0 |
| **8** | 10 | 0 |
| **9** | 4 | 0 |
| **10** | 21 | 0 |
| **11** | 18 | 0 |
| **12** | 9 | 0 |
| **13** | 13 | 0 |
| **14** | 8 | 0 |
| **15** | 7 | 0 |
| **16** | 13 | 0 |
| **17** | 9 | 0 |
| **18** | 2 | 0 |
| **19** | 6 | 0 |
| **20** | 3 | 0 |
| **21** | 0 | 0 |
| **22** | 6 | 0 |
| **X** | 1 | 144 |

Table S9: Distribution of dDHPs significantly associated with human brain development split by autosomal chromosome and direction of effect.

| **Chromosome** | **Total probes** | **Significant**  **(*P* < 5E-05)**  **probes (%)** | **Enrichment**  **(95% CI)** | ***P*-value** | **Hyper-**  **hydroxymethylated**  **(%)** | **Hypo-**  **hydroxymethylated**  **(%)** | **Proportion hyper-**  **hydroxymethylated**  **(95% CI)** | ***P*-value** |
| --- | --- | --- | --- | --- | --- | --- | --- | --- |
| 1 | 29611 | 249 (0.84) | 1.15 (1.01 - 1.32) | 3.58E-02 | 107 (42.97) | 142 (57.03) | 0.75 (0.52 - 1.09) | 1.27E-01 |
| 2 | 22286 | 196 (0.88) | 1.21 (1.04 - 1.40) | 1.34E-02 | 78 (39.80) | 118 (60.20) | 0.66 (0.43 - 1.01) | 5.35E-02 |
| 3 | 15588 | 123 (0.79) | 1.08 (0.89 - 1.30) | 3.86E-01 | 51 (41.46) | 72 (58.54) | 0.71 (0.41 - 1.21) | 2.01E-01 |
| 4 | 12712 | 92 (0.72) | 0.99 (0.80 - 1.22) | 1.00E+00 | 35 (38.04) | 57 (61.96) | 0.62 (0.33 - 1.15) | 1.37E-01 |
| 5 | 15108 | 117 (0.77) | 1.06 (0.87 - 1.28) | 5.25E-01 | 46 (39.32) | 71 (60.68) | 0.65 (0.37 - 1.13) | 1.15E-01 |
| 6 | 22664 | 142 (0.63) | 0.86 (0.72 - 1.02) | 8.01E-02 | 69 (48.59) | 73 (51.41) | 0.95 (0.58 - 1.55) | 9.06E-01 |
| 7 | 19216 | 134 (0.70) | 0.96 (0.80 - 1.14) | 6.61E-01 | 40 (29.85) | 94 (70.15) | 0.43 (0.25 - 0.72) | 1.14E-03 |
| 8 | 13604 | 109 (0.80) | 1.10 (0.90 - 1.33) | 3.29E-01 | 53 (48.62) | 56 (51.38) | 0.95 (0.54 - 1.67) | 8.92E-01 |
| 9 | 6515 | 50 (0.77) | 1.05 (0.78 - 1.39) | 7.13E-01 | 25 (50.00) | 25 (50.00) | 1.00 (0.42 - 2.36) | 1.00E+00 |
| 10 | 15932 | 104 (0.65) | 0.89 (0.73 - 1.09) | 2.92E-01 | 40 (38.46) | 64 (61.54) | 0.63 (0.35 - 1.12) | 1.24E-01 |
| 11 | 18885 | 124 (0.66) | 0.90 (0.74 - 1.08) | 2.69E-01 | 46 (37.10) | 78 (62.90) | 0.59 (0.34 - 1.01) | 5.45E-02 |
| 12 | 15100 | 92 (0.61) | 0.83 (0.67 - 1.03) | 9.41E-02 | 36 (39.13) | 56 (60.87) | 0.64 (0.34 - 1.20) | 1.82E-01 |
| 13 | 7985 | 78 (0.98) | 1.34 (1.06 - 1.68) | 1.39E-02 | 30 (38.46) | 48 (61.54) | 0.63 (0.31 - 1.24) | 1.97E-01 |
| 14 | 9377 | 87 (0.93) | 1.27 (1.02 - 1.58) | 3.14E-02 | 27 (31.03) | 60 (68.97) | 0.45 (0.23 - 0.88) | 1.33E-02 |
| 15 | 9385 | 72 (0.77) | 1.05 (0.82 - 1.33) | 6.66E-01 | 36 (50.00) | 36 (50.00) | 1.00 (0.49 - 2.02) | 1.00E+00 |
| 16 | 14400 | 84 (0.58) | 0.80 (0.63 - 0.99) | 4.38E-02 | 37 (44.05) | 47 (55.95) | 0.79 (0.41 - 1.51) | 5.37E-01 |
| 17 | 17764 | 131 (0.74) | 1.01 (0.84 - 1.21) | 8.92E-01 | 66 (50.38) | 65 (49.62) | 1.02 (0.61 - 1.70) | 1.00E+00 |
| 18 | 3451 | 24 (0.70) | 0.95 (0.61 - 1.42) | 9.20E-01 | 8 (33.33) | 16 (66.67) | 0.51 (0.13 - 1.86) | 3.80E-01 |
| 19 | 15100 | 68 (0.45) | 0.62 (0.48 - 0.78) | 3.08E-05 | 31 (45.59) | 37 (54.41) | 0.84 (0.40 - 1.73) | 7.31E-01 |
| 20 | 6424 | 36 (0.56) | 0.77 (0.54 - 1.07) | 1.19E-01 | 16 (44.44) | 20 (55.56) | 0.80 (0.29 - 2.23) | 8.14E-01 |
| 21 | 2771 | 22 (0.79) | 1.09 (0.68 - 1.66) | 6.53E-01 | 7 (31.82) | 15 (68.18) | 0.47 (0.11 - 1.86) | 3.58E-01 |
| 22 | 5094 | 47 (0.92) | 1.27 (0.93 - 1.69) | 1.15E-01 | 19 (40.43) | 28 (59.57) | 0.68 (0.28 - 1.67) | 4.08E-01 |

|  | **Total probes** | **Mean 5hmC (%) (SD)** | **Significant**  **(*P* < 5E-05) probes (%)** | **Enrichment**  **(95% CI)** | ***P*-value** | **Hyper-hydroxy-**  **methylated**  **(%)** | **Hypo-hydroxy-**  **methylated**  **(%)** | **Enrichment hypermethylated**  **(95% CI)** | ***P*-value** |
| --- | --- | --- | --- | --- | --- | --- | --- | --- | --- |
| *Intergenic non-CGI* | 33198 | 5.17 (5.58) | 340 (1.02) | 1.41 (1.25 - 1.58) | 1.99E-08 | 115 (33.82) | 225 (66.18) | 0.51 (0.37 - 0.70) | 2.59E-05 |
| *Intergenic CGI* | 6515 | 2.01 (4.40) | 30 (0.46) | 0.63 (0.42 - 0.90) | 9.50E-03 | 15 (50.00) | 15 (50.00) | 1.00 (0.32 - 3.10) | 1.00E+00 |
| *Intergenic shelf* | 4499 | 4.97 (5.30) | 37 (0.82) | 1.13 (0.79 - 1.56) | 4.79E-01 | 11 (29.73) | 26 (70.27) | 0.43 (0.14 - 1.22) | 9.67E-02 |
| *Intergenic shore* | 7942 | 4.21 (5.57) | 61 (0.77) | 1.05 (0.80 - 1.36) | 6.88E-01 | 21 (34.43) | 40 (65.57) | 0.53 (0.24 - 1.16) | 9.90E-02 |
| *Distal promoter non-CGI* | 3198 | 4.43 (5.24) | 29 (0.91) | 1.25 (0.83 - 1.80) | 2.49E-01 | 16 (55.17) | 13 (44.83) | 1.23 (0.38 - 3.95) | 7.93E-01 |
| *Distal promoter CGI* | 2265 | 1.45 (3.73) | 12 (0.53) | 0.72 (0.37 - 1.27) | 3.20E-01 | 10 (83.33) | 2 (16.67) | 4.66 (0.58 - 62.02) | 1.93E-01 |
| *Distal promoter shelf* | 6493 | 4.88 (5.04) | 31 (0.48) | 0.65 (0.44 - 0.93) | 1.75E-02 | 21 (67.74) | 10 (32.26) | 2.07 (0.66 - 6.77) | 1.98E-01 |
| *Distal promoter shore* | 3705 | 3.64 (5.51) | 40 (1.08) | 1.49 (1.06 - 2.03) | 1.94E-02 | 23 (57.50) | 17 (42.50) | 1.35 (0.51 - 3.58) | 6.54E-01 |
| *Proximal promoter non-CGI* | 25654 | 4.39 (5.23) | 184 (0.72) | 0.98 (0.84 - 1.14) | 8.78E-01 | 83 (45.11) | 101 (54.89) | 0.82 (0.53 - 1.26) | 4.04E-01 |
| *Proximal promoter CGI* | 22639 | 0.79 (3.10) | 54 (0.24) | 0.33 (0.24 - 0.43) | 1.48E-22 | 36 (66.67) | 18 (33.33) | 1.99 (0.86 - 4.69) | 1.18E-01 |
| *Proximal promoter shelf* | 3009 | 4.15 (5.18) | 24 (0.80) | 1.09 (0.70 - 1.64) | 6.66E-01 | 10 (41.67) | 14 (58.33) | 0.72 (0.20 - 2.58) | 7.72E-01 |
| *Proximal promoter shore* | 31116 | 3.78 (5.72) | 200 (0.64) | 0.88 (0.76 - 1.02) | 9.10E-02 | 80 (40.00) | 120 (60.00) | 0.67 (0.44 - 1.01) | 5.60E-02 |
| *Gene body non-CGI* | 67698 | 5.56 (5.60) | 633 (0.94) | 1.28 (1.17 - 1.40) | 6.81E-08 | 253 (39.97) | 380 (60.03) | 0.67 (0.53 - 0.84) | 3.68E-04 |
| *Gene body CGI* | 23163 | 2.15 (4.45) | 74 (0.32) | 0.44 (0.34 - 0.55) | 1.87E-15 | 36 (48.65) | 38 (51.35) | 0.95 (0.47 - 1.90) | 1.00E+00 |
| *Gene body shelf* | 20312 | 5.03 (5.42) | 159 (0.78) | 1.07 (0.91 - 1.26) | 3.95E-01 | 71 (44.65) | 88 (55.35) | 0.81 (0.51 - 1.29) | 3.69E-01 |
| *Gene body shore* | 30911 | 4.00 (5.45) | 218 (0.71) | 0.97 (0.84 - 1.11) | 6.73E-01 | 76 (34.86) | 142 (65.14) | 0.54 (0.36 - 0.80) | 1.89E-03 |
| *Downstream region non-CGI* | 2474 | 4.96 (5.43) | 27 (1.09) | 1.50 (0.99 - 2.20) | 4.31E-02 | 12 (44.44) | 15 (55.56) | 0.80 (0.24 - 2.69) | 7.86E-01 |
| *Downstream region CGI* | 1285 | 2.06 (4.15) | 6 (0.47) | 0.64 (0.23 - 1.40) | 3.25E-01 | 5 (83.33) | 1 (16.67) | 4.34 (0.22 - 313.04) | 5.45E-01 |
| *Downstream region shelf* | 1236 | 4.73 (5.28) | 10 (0.81) | 1.11 (0.53 - 2.05) | 7.35E-01 | 3 (30.00) | 7 (70.00) | 0.45 (0.05 - 3.67) | 6.50E-01 |
| *Downstream region shore* | 1660 | 3.63 (5.14) | 12 (0.72) | 0.99 (0.51 - 1.74) | 1.00E+00 | 6 (50.00) | 6 (50.00) | 1.00 (0.15 - 6.50) | 1.00E+00 |

Table S10: The distribution of dDHPs across genic features stratified by CpG density. See also Fig. S7.

Table S11: The majority of ENCODE transcription factor binding-sites (TFBSs) are characterized by a relative depletion of dDHPs. TFBSs displaying a significant (*P* < 0.05) under or over-enrichment of significant dDHPs are shown, as well as direction of effect. TFBSs were derived from recent publications (ENCODE Project Consortium 2012; Slieker et al. 2013).

| **Transcription Factor Binding Site** | **Total**  **probes** | **Mean 5hmC (%) (SD)** | **Significant**  **(*P* < 5E-05) probes (%)** | **Enrichment**  **(95% CI)** | ***P*-value** | **Hyper-hydroxy-methylated**  **(%)** | **Hypo-hydroxy-methylated**  **(%)** | **Enrichment hyper-hydroxymethylated**  **(95% CI)** | ***P*-value** |
| --- | --- | --- | --- | --- | --- | --- | --- | --- | --- |
| **Under-enriched for dDHPs** |  |  |  |  |  |  |  |  |  |
| *ATF3* | 1894 | 1.01 (3.66) | 5 (0.26) | 0.36 (0.12 - 0.84) | 1.35E-02 | 1 (20.00) | 4 (80.00) | 0.30 (0.00 - 9.08) | 5.24E-01 |
| *BRCA1 (C-1863)* | 2007 | 1.06 (3.98) | 5 (0.25) | 0.34 (0.11 - 0.80) | 7.66E-03 | 3 (60.00) | 2 (40.00) | 1.43 (0.05 - 39.79) | 1.00E+00 |
| *CCNT2* | 6866 | 1.33 (3.78) | 31 (0.45) | 0.62 (0.42 - 0.88) | 6.01E-03 | 16 (51.61) | 15 (48.39) | 1.07 (0.35 - 3.27) | 1.00E+00 |
| *CHD2 (N-1250)* | 4735 | 0.94 (3.61) | 12 (0.25) | 0.35 (0.18 - 0.61) | 1.90E-05 | 7 (58.33) | 5 (41.67) | 1.38 (0.21 - 9.33) | 1.00E+00 |
| *CtBP2* | 1661 | 1.28 (3.59) | 4 (0.24) | 0.33 (0.09 - 0.84) | 1.31E-02 | 3 (75.00) | 1 (25.00) | 2.60 (0.08 - 234.45) | 1.00E+00 |
| *CTCF (C-20)* | 5398 | 2.00 (4.73) | 25 (0.46) | 0.63 (0.41 - 0.94) | 1.88E-02 | 13 (52.00) | 12 (48.00) | 1.08 (0.31 - 3.83) | 1.00E+00 |
| *CTCF (SC-5916)* | 7014 | 2.19 (4.78) | 31 (0.44) | 0.60 (0.41 - 0.86) | 3.39E-03 | 19 (61.29) | 12 (38.71) | 1.57 (0.51 - 4.95) | 4.44E-01 |
| *E2F1* | 1934 | 0.58 (2.97) | 0 (0.00) | 0.00 (0.00 - 0.26) | 1.27E-06 | 0 (NA) | 0 (NA) | 0.00 (0.00 - Inf) | 1.00E+00 |
| *E2F4* | 3212 | 0.51 (3.11) | 3 (0.09) | 0.13 (0.03 - 0.37) | 3.05E-07 | 2 (66.67) | 1 (33.33) | 1.73 (0.01 - 233.82) | 1.00E+00 |
| *E2F6* | 6408 | 1.57 (4.19) | 25 (0.39) | 0.53 (0.34 - 0.79) | 7.60E-04 | 9 (36.00) | 16 (64.00) | 0.57 (0.15 - 2.03) | 3.93E-01 |
| *E2F6 (H-50)* | 8742 | 1.69 (4.31) | 32 (0.37) | 0.50 (0.34 - 0.71) | 2.05E-05 | 13 (40.63) | 19 (59.38) | 0.69 (0.23 - 2.05) | 6.16E-01 |
| *Egr-1* | 9486 | 1.70 (4.25) | 43 (0.45) | 0.62 (0.45 - 0.84) | 1.06E-03 | 16 (37.21) | 27 (62.79) | 0.60 (0.23 - 1.53) | 2.78E-01 |
| *ELF1 (SC-631)* | 13577 | 1.68 (4.29) | 74 (0.55) | 0.75 (0.58 - 0.94) | 1.27E-02 | 33 (44.59) | 41 (55.41) | 0.81 (0.40 - 1.61) | 6.22E-01 |
| *ELK4* | 3378 | 0.76 (3.69) | 13 (0.38) | 0.53 (0.28 - 0.90) | 1.43E-02 | 6 (46.15) | 7 (53.85) | 0.86 (0.14 - 5.36) | 1.00E+00 |
| *ETS1* | 3926 | 0.94 (3.57) | 17 (0.43) | 0.59 (0.34 - 0.95) | 2.88E-02 | 8 (47.06) | 9 (52.94) | 0.89 (0.18 - 4.27) | 1.00E+00 |
| *GABP* | 8165 | 1.29 (3.92) | 28 (0.34) | 0.47 (0.31 - 0.68) | 1.05E-05 | 12 (42.86) | 16 (57.14) | 0.75 (0.23 - 2.44) | 7.89E-01 |
| *GTF2B* | 1121 | 0.52 (3.58) | 0 (0.00) | 0.00 (0.00 - 0.45) | 5.67E-04 | 0 (NA) | 0 (NA) | 0.00 (0.00 - Inf) | 1.00E+00 |
| *GTF2F1 (RAP-74)* | 4550 | 1.42 (4.37) | 18 (0.40) | 0.54 (0.32 - 0.86) | 6.11E-03 | 8 (44.44) | 10 (55.56) | 0.80 (0.18 - 3.58) | 1.00E+00 |
| *HA-E2F1* | 13497 | 1.25 (3.90) | 40 (0.30) | 0.40 (0.29 - 0.55) | 8.38E-11 | 14 (35.00) | 26 (65.00) | 0.54 (0.20 - 1.45) | 2.58E-01 |
| *HEY1* | 11222 | 1.35 (4.03) | 35 (0.31) | 0.43 (0.30 - 0.59) | 1.27E-08 | 17 (48.57) | 18 (51.43) | 0.95 (0.33 - 2.69) | 1.00E+00 |
| *HMGN3* | 5658 | 1.16 (3.50) | 20 (0.35) | 0.48 (0.29 - 0.75) | 4.52E-04 | 11 (55.00) | 9 (45.00) | 1.22 (0.30 - 5.04) | 1.00E+00 |
| *HNF4A (H-171)* | 2821 | 3.14 (4.92) | 11 (0.39) | 0.53 (0.27 - 0.96) | 3.33E-02 | 5 (45.45) | 6 (54.55) | 0.84 (0.11 - 6.29) | 1.00E+00 |
| *IRF1* | 6262 | 0.74 (3.40) | 17 (0.27) | 0.37 (0.22 - 0.59) | 2.35E-06 | 10 (58.82) | 7 (41.18) | 1.41 (0.29 - 7.00) | 7.32E-01 |
| *IRF3* | 1106 | -0.11 (2.73) | 1 (0.09) | 0.12 (0.00 - 0.69) | 6.52E-03 | 1 (100.00) | 0 (0.00) | 0.00 (0.00 - Inf) | 1.00E+00 |
| *Mxi1 (bHLH)* | 4907 | 1.33 (4.12) | 23 (0.47) | 0.64 (0.40 - 0.97) | 3.33E-02 | 13 (56.52) | 10 (43.48) | 1.29 (0.34 - 4.92) | 7.68E-01 |
| *NF-YA* | 2489 | 0.62 (3.40) | 1 (0.04) | 0.05 (0.00 - 0.31) | 5.64E-07 | 1 (100.00) | 0 (0.00) | 0.00 (0.00 - Inf) | 1.00E+00 |
| *NF-YB* | 2665 | 0.66 (3.39) | 1 (0.04) | 0.05 (0.00 - 0.29) | 1.24E-07 | 1 (100.00) | 0 (0.00) | 0.00 (0.00 - Inf) | 1.00E+00 |
| *NFKB* | 10382 | 2.02 (4.63) | 55 (0.53) | 0.72 (0.54 - 0.95) | 1.82E-02 | 24 (43.64) | 31 (56.36) | 0.78 (0.34 - 1.76) | 5.67E-01 |
| *Nrf1* | 3155 | 0.51 (3.09) | 4 (0.13) | 0.17 (0.05 - 0.44) | 2.46E-06 | 4 (100.00) | 0 (0.00) | Inf (0.20 - Inf) | 4.29E-01 |
| *PAX5-C20* | 5819 | 1.90 (4.41) | 25 (0.43) | 0.59 (0.38 - 0.87) | 6.07E-03 | 14 (56.00) | 11 (44.00) | 1.27 (0.36 - 4.53) | 7.78E-01 |
| *Pol2* | 38610 | 2.23 (4.70) | 228 (0.59) | 0.81 (0.70 - 0.93) | 2.03E-03 | 93 (40.79) | 135 (59.21) | 0.69 (0.47 - 1.01) | 5.98E-02 |
| *Pol2-4H8* | 20627 | 2.07 (4.58) | 105 (0.51) | 0.70 (0.57 - 0.85) | 1.67E-04 | 46 (43.81) | 59 (56.19) | 0.78 (0.44 - 1.39) | 4.07E-01 |
| *Pol2(phosphoS2)* | 4803 | 1.01 (3.72) | 16 (0.33) | 0.45 (0.26 - 0.74) | 5.54E-04 | 5 (31.25) | 11 (68.75) | 0.47 (0.08 - 2.37) | 4.73E-01 |
| *RFX5 (N-494)* | 4939 | 1.61 (4.52) | 20 (0.40) | 0.55 (0.34 - 0.86) | 5.13E-03 | 8 (40.00) | 12 (60.00) | 0.67 (0.16 - 2.76) | 7.51E-01 |
| *SETDB1* | 2022 | 2.43 (5.17) | 6 (0.30) | 0.41 (0.15 - 0.88) | 1.73E-02 | 3 (50.00) | 3 (50.00) | 1.00 (0.06 - 15.64) | 1.00E+00 |
| *Sin3Ak-20* | 8326 | 1.12 (3.80) | 21 (0.25) | 0.34 (0.21 - 0.53) | 1.06E-08 | 13 (61.90) | 8 (38.10) | 1.61 (0.40 - 6.73) | 5.36E-01 |
| *SIX5* | 2230 | 0.17 (2.88) | 2 (0.09) | 0.12 (0.01 - 0.44) | 2.50E-05 | 1 (50.00) | 1 (50.00) | 1.00 (0.01 - 155.93) | 1.00E+00 |
| *SMC3 (ab9263)* | 6970 | 2.26 (4.82) | 35 (0.50) | 0.69 (0.48 - 0.96) | 2.64E-02 | 15 (42.86) | 20 (57.14) | 0.75 (0.26 - 2.15) | 6.32E-01 |
| *SP1* | 10314 | 1.79 (4.36) | 43 (0.42) | 0.57 (0.41 - 0.77) | 8.74E-05 | 25 (58.14) | 18 (41.86) | 1.38 (0.54 - 3.57) | 5.17E-01 |
| *SP2 (SC-643)* | 1485 | 0.16 (2.65) | 0 (0.00) | 0.00 (0.00 - 0.34) | 3.96E-05 | 0 (NA) | 0 (NA) | 0.00 (0.00 - Inf) | 1.00E+00 |
| *SRF* | 2925 | 1.75 (4.48) | 12 (0.41) | 0.56 (0.29 - 0.98) | 4.75E-02 | 6 (50.00) | 6 (50.00) | 1.00 (0.15 - 6.50) | 1.00E+00 |
| *TAF1* | 16725 | 1.25 (3.87) | 70 (0.42) | 0.57 (0.44 - 0.73) | 8.23E-07 | 35 (50.00) | 35 (50.00) | 1.00 (0.49 - 2.04) | 1.00E+00 |
| *TAF7 (SQ-8)* | 2594 | 0.57 (3.17) | 5 (0.19) | 0.26 (0.09 - 0.62) | 4.03E-04 | 3 (60.00) | 2 (40.00) | 1.43 (0.05 - 39.79) | 1.00E+00 |
| *TBP* | 16368 | 1.65 (4.38) | 70 (0.43) | 0.58 (0.45 - 0.74) | 2.18E-06 | 35 (50.00) | 35 (50.00) | 1.00 (0.49 - 2.04) | 1.00E+00 |
| *TR4* | 1560 | 0.74 (3.50) | 1 (0.06) | 0.09 (0.00 - 0.49) | 2.55E-04 | 1 (100.00) | 0 (0.00) | 0.00 (0.00 - Inf) | 1.00E+00 |
| *YY1* | 6927 | 1.32 (4.20) | 27 (0.39) | 0.53 (0.35 - 0.78) | 5.33E-04 | 10 (37.04) | 17 (62.96) | 0.59 (0.17 - 2.01) | 4.12E-01 |
| *YY1 (C-20)* | 10234 | 1.36 (4.14) | 54 (0.53) | 0.72 (0.54 - 0.95) | 1.74E-02 | 23 (42.59) | 31 (57.41) | 0.74 (0.32 - 1.69) | 5.63E-01 |
| *ZBTB33* | 1298 | 1.29 (4.09) | 3 (0.23) | 0.32 (0.06 - 0.92) | 3.13E-02 | 2 (66.67) | 1 (33.33) | 1.73 (0.01 - 233.82) | 1.00E+00 |
| *ZBTB7A (SC-34508)* | 6631 | 1.48 (3.82) | 27 (0.41) | 0.56 (0.37 - 0.81) | 1.22E-03 | 12 (44.44) | 15 (55.56) | 0.80 (0.24 - 2.69) | 7.86E-01 |
| *Znf143 (16618-1-AP)* | 4309 | 0.82 (3.62) | 5 (0.12) | 0.16 (0.05 - 0.37) | 1.71E-08 | 3 (60.00) | 2 (40.00) | 1.43 (0.05 - 39.79) | 1.00E+00 |
|  |  |  |  |  |  |  |  |  |  |
| **Enriched for dDHPs** |  |  |  |  |  |  |  |  |  |
| *BATF* | 1946 | 4.83 (5.79) | 24 (1.23) | 1.70 (1.08 - 2.54) | 1.53E-02 | 12 (50.00) | 12 (50.00) | 1.00 (0.28 - 3.58) | 1.00E+00 |
| *BCL11A* | 1712 | 4.58 (5.64) | 26 (1.52) | 2.10 (1.36 - 3.09) | 8.51E-04 | 9 (34.62) | 17 (65.38) | 0.54 (0.15 - 1.85) | 4.00E-01 |
| *c-Jun* | 5577 | 4.50 (5.67) | 57 (1.02) | 1.41 (1.06 - 1.83) | 1.41E-02 | 12 (21.05) | 45 (78.95) | 0.27 (0.11 - 0.65) | 1.63E-03 |
| *EBF* | 3700 | 3.61 (5.33) | 39 (1.05) | 1.45 (1.03 - 1.99) | 2.56E-02 | 14 (35.90) | 25 (64.10) | 0.56 (0.20 - 1.53) | 2.53E-01 |
| *eGFP-FOS* | 1535 | 4.35 (5.41) | 18 (1.17) | 1.61 (0.95 - 2.57) | 4.96E-02 | 4 (22.22) | 14 (77.78) | 0.30 (0.05 - 1.47) | 1.64E-01 |
| *ERalpha_a* | 3179 | 3.68 (5.20) | 39 (1.23) | 1.69 (1.20 - 2.32) | 2.32E-03 | 19 (48.72) | 20 (51.28) | 0.95 (0.35 - 2.55) | 1.00E+00 |
| *GATA-2* | 6967 | 4.49 (5.87) | 94 (1.35) | 1.86 (1.50 - 2.29) | 6.52E-08 | 35 (37.23) | 59 (62.77) | 0.59 (0.32 - 1.10) | 1.05E-01 |
| *GR* | 4536 | 3.99 (5.37) | 56 (1.23) | 1.70 (1.28 - 2.22) | 2.99E-04 | 20 (35.71) | 36 (64.29) | 0.56 (0.24 - 1.27) | 1.81E-01 |
| *p300* | 9119 | 3.63 (5.43) | 87 (0.95) | 1.31 (1.04 - 1.63) | 1.79E-02 | 32 (36.78) | 55 (63.22) | 0.58 (0.30 - 1.12) | 9.24E-02 |
| *p300 (N-15)* | 2195 | 4.95 (5.83) | 29 (1.32) | 1.82 (1.21 - 2.63) | 3.44E-03 | 7 (24.14) | 22 (75.86) | 0.32 (0.09 - 1.12) | 5.70E-02 |
| *PRDM1 (Val90)* | 408 | 3.64 (5.69) | 7 (1.72) | 2.38 (0.95 - 4.95) | 3.20E-02 | 1 (14.29) | 6 (85.71) | 0.19 (0.00 - 3.64) | 2.66E-01 |
| *STAT3* | 6347 | 4.27 (5.69) | 82 (1.29) | 1.78 (1.41 - 2.22) | 2.59E-06 | 21 (25.61) | 61 (74.39) | 0.35 (0.17 - 0.70) | 2.09E-03 |
| *TAL1_(SC-12984)* | 2306 | 3.87 (5.43) | 26 (1.13) | 1.55 (1.01 - 2.29) | 3.56E-02 | 10 (38.46) | 16 (61.54) | 0.63 (0.18 - 2.15) | 5.77E-01 |

**Table S12: dDHPs in genomic regions characterized by alternative transcription events.** See also **Fig. S8**.

| **Alternative transcription event** | **Total probes** | **Mean 5hmC (%) (SD)** | **Significant**  **(*P* < 5E-05) probes (%)** | **Enrichment**  **(95% CI)** | ***P*-value** | **Hyper-hydroxy-methylated**  **(%)** | **Hypo-hydroxymethylated**  **(%)** | **Enrichment hyper-hydroxymethylated**  **(95% CI)** | ***P*-value** |
| --- | --- | --- | --- | --- | --- | --- | --- | --- | --- |
| *Alternative 3’ splice site* | 2646 | 3.14 (4.90) | 9 (0.34) | 0.46 (0.21 - 0.88) | 1.51E-02 | 3 (33.33) | 6 (66.67) | 0.52 (0.05 - 5.13) | 6.37E-01 |
| *Alternative 5’ splice site* | 2630 | 3.28 (4.94) | 20 (0.76) | 1.04 (0.63 - 1.62) | 8.17E-01 | 12 (60.00) | 8 (40.00) | 1.48 (0.36 - 6.29) | 7.51E-01 |
| *Alternative first exon* | 34633 | 3.90 (5.55) | 265 (0.77) | 1.05 (0.92 - 1.19) | 4.64E-01 | 109 (41.13) | 156 (58.87) | 0.70 (0.49 - 1.00) | 4.48E-02 |
| *Alternative last exon* | 9047 | 4.79 (5.48) | 77 (0.85) | 1.17 (0.92 - 1.47) | 1.88E-01 | 40 (51.95) | 37 (48.05) | 1.08 (0.55 - 2.14) | 8.72E-01 |
| *Cassette exon* | 51206 | 4.60 (5.56) | 399 (0.78) | 1.07 (0.96 - 1.19) | 2.29E-01 | 161 (40.35) | 238 (59.65) | 0.68 (0.51 - 0.90) | 6.84E-03 |
| *Constitutive exon* | 16080 | 2.97 (4.91) | 70 (0.44) | 0.59 (0.46 - 0.76) | 4.70E-06 | 41 (58.57) | 29 (41.43) | 1.41 (0.69 - 2.91) | 3.96E-01 |
| *Exon isoforms* | 137 | 2.74 (4.99) | 1 (0.73) | 1.00 (0.03 - 5.68) | 1.00E+00 | 0 (0.00) | 1 (100.00) | 0.00 (0.00 - Inf) | 1.00E+00 |
| *Intron isoforms* | 20290 | 3.98 (5.46) | 148 (0.73) | 1.00 (0.84 - 1.18) | 1.00E+00 | 58 (39.19) | 90 (60.81) | 0.65 (0.40 - 1.05) | 7.92E-02 |
| *Intron retention* | 11890 | 3.21 (4.87) | 78 (0.66) | 0.90 (0.71 - 1.13) | 3.79E-01 | 41 (52.56) | 37 (47.44) | 1.11 (0.56 - 2.18) | 8.73E-01 |
| *Mutually exclusive exon* | 11295 | 4.92 (5.65) | 80 (0.71) | 0.97 (0.77 - 1.21) | 8.66E-01 | 38 (47.50) | 42 (52.50) | 0.91 (0.46 - 1.76) | 8.74E-01 |

Table S13: Mean DNA hydroxymethylation (%) by chromosome for all samples, and males and females separately

|  |  | **All samples**  **(n = 71)** | **Females**  **(n = 36)** | **Males**  **(n = 35)** |
| --- | --- | --- | --- | --- |
| **Chromosome** | **Total probes** | **Mean 5hmC (%) (SD)** | **Mean 5hmC (%) (SD)** | **Mean 5hmC (%) (SD)** |
| 1 | 29611 | 4.30 (5.44) | 4.35 (5.49) | 4.26 (5.38) |
| 2 | 22286 | 4.53 (5.57) | 4.59 (5.62) | 4.47 (5.51) |
| 3 | 15588 | 4.64 (5.57) | 4.69 (5.62) | 4.58 (5.51) |
| 4 | 12712 | 4.36 (5.55) | 4.40 (5.59) | 4.32 (5.51) |
| 5 | 15108 | 4.31 (5.60) | 4.36 (5.64) | 4.26 (5.55) |
| 6 | 22664 | 4.02 (5.42) | 4.06 (5.46) | 3.99 (5.37) |
| 7 | 19216 | 4.29 (5.51) | 4.34 (5.56) | 4.24 (5.47) |
| 8 | 13604 | 4.52 (5.53) | 4.57 (5.58) | 4.46 (5.48) |
| 9 | 6515 | 4.23 (5.42) | 4.28 (5.48) | 4.19 (5.37) |
| 10 | 15932 | 4.49 (5.60) | 4.55 (5.65) | 4.44 (5.55) |
| 11 | 18885 | 4.06 (5.30) | 4.10 (5.36) | 4.02 (5.25) |
| 12 | 15100 | 4.12 (5.45) | 4.17 (5.49) | 4.07 (5.39) |
| 13 | 7985 | 4.68 (5.67) | 4.74 (5.72) | 4.61 (5.61) |
| 14 | 9377 | 4.21 (5.47) | 4.25 (5.52) | 4.16 (5.41) |
| 15 | 9385 | 4.31 (5.47) | 4.36 (5.53) | 4.26 (5.41) |
| 16 | 14400 | 3.70 (5.20) | 3.74 (5.25) | 3.66 (5.15) |
| 17 | 17764 | 3.80 (5.23) | 3.84 (5.28) | 3.76 (5.17) |
| 18 | 3451 | 4.01 (5.49) | 4.06 (5.54) | 3.96 (5.44) |
| 19 | 15100 | 2.80 (4.71) | 2.81 (4.73) | 2.80 (4.69) |
| 20 | 6424 | 3.69 (5.15) | 3.71 (5.20) | 3.66 (5.11) |
| 21 | 2771 | 3.85 (5.19) | 3.87 (5.22) | 3.83 (5.17) |
| 22 | 5094 | 3.89 (5.16) | 3.92 (5.19) | 3.86 (5.12) |
| X | 8838 | 2.00 (5.10) | 1.40 (4.63) | 2.61 (5.47) |
| Y | 14 | - | - | 0.30 (4.25) |
| **All probes** | 307,824 | 4.09 (5.43) | 4.12 (5.48) | 4.07 (5.39) |
| **Autosomal probes** | 298,972 | 4.16 (5.43) | 4.20 (5.48) | 4.11 (5.38) |

Table S15: Co-hydroxymethylation modules in the developing fetal brain. A total of 32 modules were identified by WGCNA analysis. Modules are shown alongside their assigned colour and ordered by module size. Shown for each module is the module correlation with DPC and sex. DPC = days post conception. See also Fig. 4a.

| **Module** | **Colour label** | **Probes** | **Genes associated with module** | **Un-annotated probes** | **XY probes (%)** | **Correlation with DPC (*P-value*)** | **Correlation with sex (*P-value*)** |
| --- | --- | --- | --- | --- | --- | --- | --- |
| 1 | turquoise | 54543 | 15199 | 15323 | 1089 (2%) | 0.072 (0.55) | -0.015 (0.9) |
| 2 | blue | 40583 | 9919 | 11580 | 109 (0.27%) | -0.48 (2.5e-05) | -0.058 (0.63) |
| 3 | brown | 12354 | 5483 | 3580 | 195 (1.6%) | 0.6 (3.4e-08) | -0.034 (0.78) |
| 4 | yellow | 12007 | 7174 | 2351 | 372 (3.1%) | 0.27 (0.022) | 0.23 (0.056) |
| 5 | green | 9378 | 5930 | 2073 | 280 (3%) | 0.42 (0.00023) | 0.0072 (0.95) |
| 6 | red | 8642 | 4424 | 2508 | 40 (0.46%) | -0.43 (0.00017) | -0.021 (0.86) |
| 7 | black | 6593 | 2885 | 2180 | 21 (0.32%) | -0.47 (3.3e-05) | -0.15 (0.22) |
| 8 | pink | 3409 | 2788 | 603 | 66 (1.9%) | 0.21 (0.075) | -0.032 (0.79) |
| 9 | magenta | 2729 | 1879 | 694 | 144 (5.3%) | 0.17 (0.15) | -0.15 (0.2) |
| 10 | purple | 2670 | 1928 | 609 | 189 (7.1%) | 0.14 (0.24) | -0.12 (0.32) |
| 11 | greenyellow | 2406 | 1626 | 705 | 59 (2.5%) | 0.15 (0.2) | 0.052 (0.67) |
| 12 | tan | 2387 | 2097 | 392 | 68 (2.8%) | 0.16 (0.19) | -0.093 (0.44) |
| 13 | salmon | 2382 | 1726 | 659 | 60 (2.5%) | -0.005 (0.97) | -0.08 (0.51) |
| 14 | cyan | 2304 | 1516 | 592 | 33 (1.4%) | 0.079 (0.51) | 0.042 (0.73) |
| 15 | midnightblue | 2255 | 1391 | 579 | 10 (0.44%) | 0.32 (0.0066) | -0.081 (0.5) |
| 16 | lightcyan | 2246 | 931 | 487 | 1841 (82%) | 0.077 (0.52) | 0.95 (2.6e-35) |
| 17 | grey60 | 2240 | 1619 | 491 | 4 (0.18%) | 0.021 (0.86) | -0.17 (0.16) |
| 18 | lightgreen | 2227 | 1319 | 671 | 28 (1.3%) | -0.42 (3e-04) | 0.16 (0.19) |
| 19 | lightyellow | 2119 | 1260 | 646 | 17 (0.8%) | -0.098 (0.41) | 0.051 (0.68) |
| 20 | royalblue | 2059 | 1307 | 499 | 29 (1.4%) | -0.043 (0.72) | 0.14 (0.26) |
| 21 | darkred | 2030 | 1253 | 493 | 22 (1.1%) | -0.13 (0.29) | 0.071 (0.56) |
| 22 | darkgreen | 2028 | 1228 | 623 | 5 (0.25%) | -0.075 (0.54) | 0.036 (0.76) |
| 23 | darkturquoise | 1998 | 1421 | 530 | 27 (1.4%) | 0.2 (0.091) | -0.031 (0.8) |
| 24 | darkgrey | 1994 | 1376 | 493 | 17 (0.85%) | -0.27 (0.025) | -0.12 (0.33) |
| 25 | orange | 1994 | 1419 | 538 | 26 (1.3%) | 0.081 (0.5) | 0.071 (0.55) |
| 26 | darkorange | 1959 | 1355 | 613 | 77 (3.9%) | 0.16 (0.18) | 0.14 (0.25) |
| 27 | white | 1909 | 1082 | 447 | 3 (0.16%) | -0.16 (0.18) | -0.18 (0.12) |
| 28 | skyblue | 1907 | 1364 | 464 | 17 (0.89%) | 0.054 (0.66) | -0.0055 (0.96) |
| 29 | saddlebrown | 1725 | 1289 | 474 | 81 (4.7%) | 0.34 (0.0038) | 0.17 (0.16) |
| 30 | steelblue | 1453 | 968 | 395 | 7 (0.48%) | -0.18 (0.13) | -0.12 (0.3) |
| 31 | paleturquoise | 1270 | 928 | 342 | 58 (4.6%) | 0.49 (1.3e-05) | 0.013 (0.91) |
| 32 | violet | 1115 | 860 | 255 | 6 (0.54%) | -0.28 (0.017) | -0.039 (0.75) |

Table S17: Hydroxymethylation QTLs in human fetal brain. See also Fig. 5.

| ***P*-value threshold** |  | **hmQTL** | **mQTL** |
| --- | --- | --- | --- |
| 1.00E-10 | **nQTL** | 305 | 35450 |
|  | **nProbes** | 67 | 1427 |
|  | **nSNPs** | 305 | 28548 |
|  | **Median effect** | 4.81 | 8.33 |
|  | **% cis** | 42.62 | 82.30 |
|  | **% same chromosome** | 57.70 | 89.76 |
| 1.00E-11 | **nQTL** | 77 | 24286 |
|  | **nProbes** | 10 | 860 |
|  | **nSNPs** | 77 | 19754 |
|  | **Median effect** | 7.08 | 8.93 |
|  | **% cis** | 80.52 | 86.30 |
|  | **% same chromosome** | 92.21 | 91.54 |
| 1.00E-12 | **nQTL** | 29 | 17669 |
|  | **nProbes** | 5 | 627 |
|  | **nSNPs** | 29 | 15036 |
|  | **Median effect** | 6.99 | 9.93 |
|  | **% cis** | 65.52 | 85.71 |
|  | **% same chromosome** | 96.55 | 90.93 |
| Bonferroni significant  (2.2E-13 for hmQTL;  1.6E-13 for mQTL) | **nQTL** | 23 | 13480 |
|  | **nProbes** | 4 | 493 |
|  | **nSNPs** | 23 | 11751 |
|  | **Median effect** | 6.99 | 9.86 |
|  | **% cis** | 73.91 | 84.18 |
|  | **% same chromosome** | 100.00 | 89.71 |

Table S19: Human fetal brain samples included in this study. M = male; F = female. Age is given in days post-conception (DPC) as determined by Carnegie staging for embryonic samples defined as ≤56 days post-conception, and foot and knee to heel length measurements in the case of fetal samples defined as ≥57 days post-conception. HDBR = the Human Developmental Biology Resource (HDBR) fetal tissue bank; MRC = the MRC London Brainbank for Neurodegenerative Diseases at the Institute of Psychiatry, King’s College London (see Methods). No additional phenotypic or demographic data was available for these samples. The DPC and sex distribution of the samples used in this study is shown in Fig. S14.

| **Sample ID** | **Sex** | **Source** | **Age** |  | **Sample ID** | **Sex** | **Source** | **Age** |
| --- | --- | --- | --- | --- | --- | --- | --- | --- |
| 1 | M | HDBR | 23 |  | 37 | F | HDBR | 94 |
| 2 | F | HDBR | 37 |  | 38 | M | HDBR | 96 |
| 3 | F | HDBR | 44 |  | 39 | F | HDBR | 96 |
| 4 | F | HDBR | 47 |  | 40 | F | HDBR | 98 |
| 5 | M | HDBR | 49 |  | 41 | M | HDBR | 99 |
| 6 | M | HDBR | 53 |  | 42 | F | HDBR | 100 |
| 7 | F | HDBR | 56 |  | 43 | F | HDBR | 102 |
| 8 | M | HDBR | 58 |  | 44 | M | HDBR | 103 |
| 9 | F | HDBR | 61 |  | 45 | F | HDBR | 104 |
| 10 | M | HDBR | 62 |  | 46 | M | HDBR | 106 |
| 11 | F | HDBR | 63 |  | 47 | F | HDBR | 107 |
| 12 | M | HDBR | 64 |  | 48 | M | HDBR | 108 |
| 13 | F | HDBR | 65 |  | 49 | M | HDBR | 110 |
| 14 | F | HDBR | 67 |  | 50 | F | HDBR | 110 |
| 15 | M | HDBR | 68 |  | 51 | F | MRC | 112 |
| 16 | F | HDBR | 70 |  | 52 | M | HDBR | 112 |
| 17 | M | HDBR | 70 |  | 53 | F | HDBR | 114 |
| 18 | M | HDBR | 72 |  | 54 | M | HDBR | 114 |
| 19 | M | HDBR | 74 |  | 55 | M | HDBR | 116 |
| 20 | F | HDBR | 74 |  | 56 | M | HDBR | 118 |
| 21 | M | HDBR | 76 |  | 57 | F | HDBR | 118 |
| 22 | F | HDBR | 76 |  | 58 | M | HDBR | 120 |
| 23 | F | HDBR | 78 |  | 59 | F | HDBR | 120 |
| 24 | M | HDBR | 80 |  | 60 | M | HDBR | 123 |
| 25 | F | HDBR | 80 |  | 61 | F | HDBR | 124 |
| 26 | M | HDBR | 82 |  | 62 | F | HDBR | 127 |
| 27 | F | HDBR | 83 |  | 63 | F | HDBR | 129 |
| 28 | M | HDBR | 84 |  | 64 | M | MRC | 133 |
| 29 | M | HDBR | 86 |  | 65 | M | MRC | 140 |
| 30 | F | HDBR | 86 |  | 66 | F | MRC | 140 |
| 31 | F | HDBR | 88 |  | 67 | M | HDBR | 153 |
| 32 | F | HDBR | 90 |  | 68 | F | MRC | 154 |
| 33 | M | HDBR | 90 |  | 69 | F | MRC | 161 |
| 34 | F | HDBR | 92 |  | 70 | M | HDBR | 169 |
| 35 | M | HDBR | 92 |  | 71 | M | HDBR | 184 |
| 36 | M | HDBR | 94 |  |  |  |  |  |

Table S20: Cross-reactive and SNP-affected probes excluded during quality control of DNA hydroxymethylation data

| **Probe type** | **Criteria** | **(n)** | **Source** |
| --- | --- | --- | --- |
| SNP probes (rs probes) | All | 64 | Illumina annotation |
| Non-specific cg probes | All | 29,233 | Chen *et al*., 2013 |
| Non-specific ch probes | All | 1,736 | Chen *et al*., 2013 |
| Non-specific cg probes | All | 41,937 | Price *et al*., 2013 |
| SNP probes | Within ≤ 10bp of SBE site, and AF ≥ 0.05 | 14,090 | Chen *et al*., 2013 |
| SNP probes | Within ≤ 10bp of SBE site, and AF ≥ 0.05 | 17,944 | Illumina annotation |
| **Total unique probes removed:** | | **58,559** | |
