# Supplementary material for: 5-hydroxymethylcytosine is highly dynamic across human fetal brain development
Source: BMC Genomics. 2017 Sep 18;18:738. doi: 10.1186/s12864-017-4091-x (PMC5604137; doi:10.1186/s12864-017-4091-x)
Supplement: Supplementary file 2 — Supplementary Figures. (DOC 3539 kb) [file 12864_2017_4091_MOESM2_ESM.doc]

Figure S1: The distribution of 5hmC across CpG island and gene features. Shown for the n = 298,972 autosomal probes included in the final dataset are: A) boxplots of 5hmC by CpG island feature, demonstrating an inverse relationship between CpG density and levels of 5hmC, and B) boxplots of 5hmC by genic feature, demonstrating an enrichment in the gene body and 3’UTR. See also Table S3.

**A)**

**
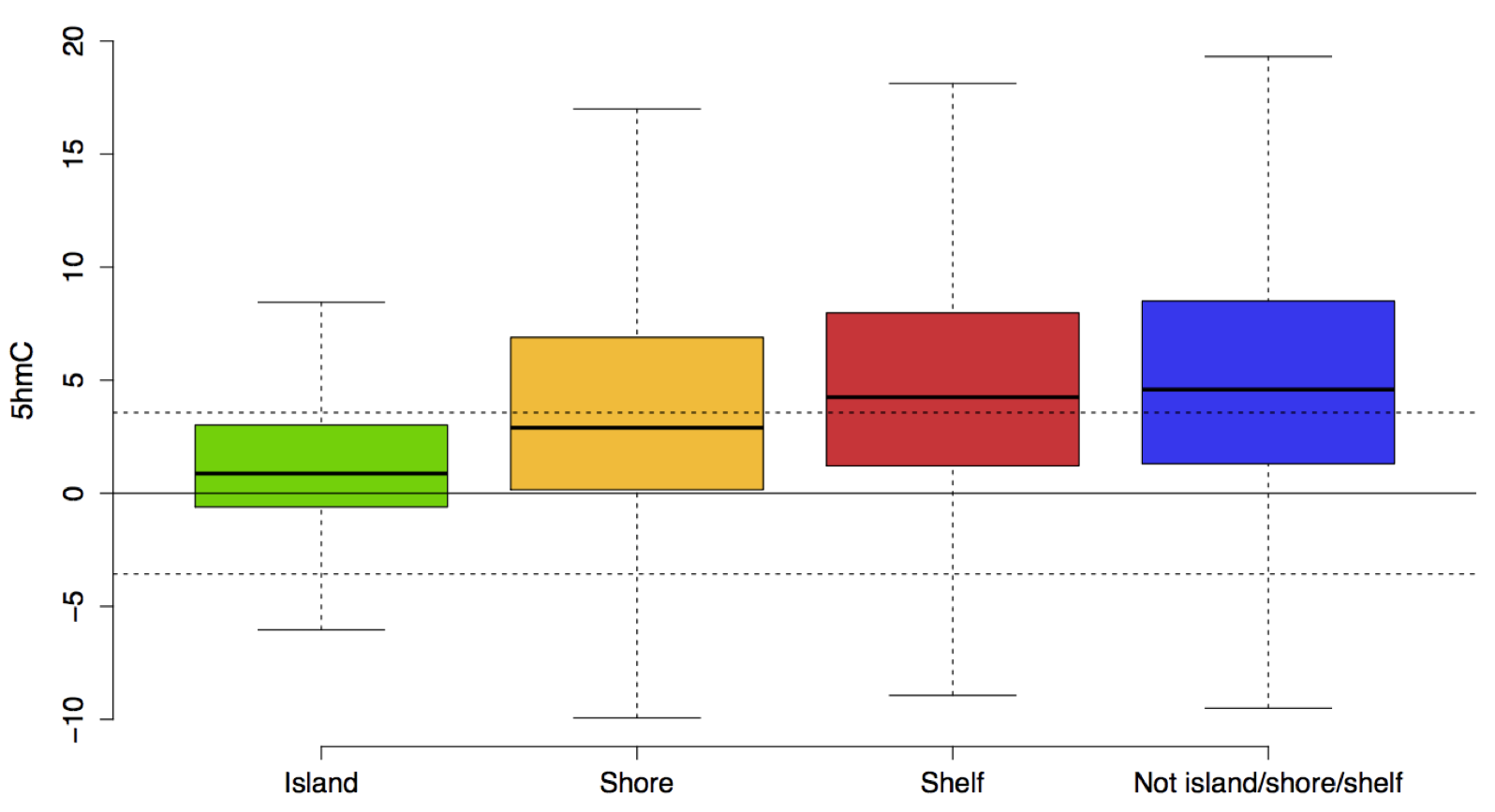
**

**B)**


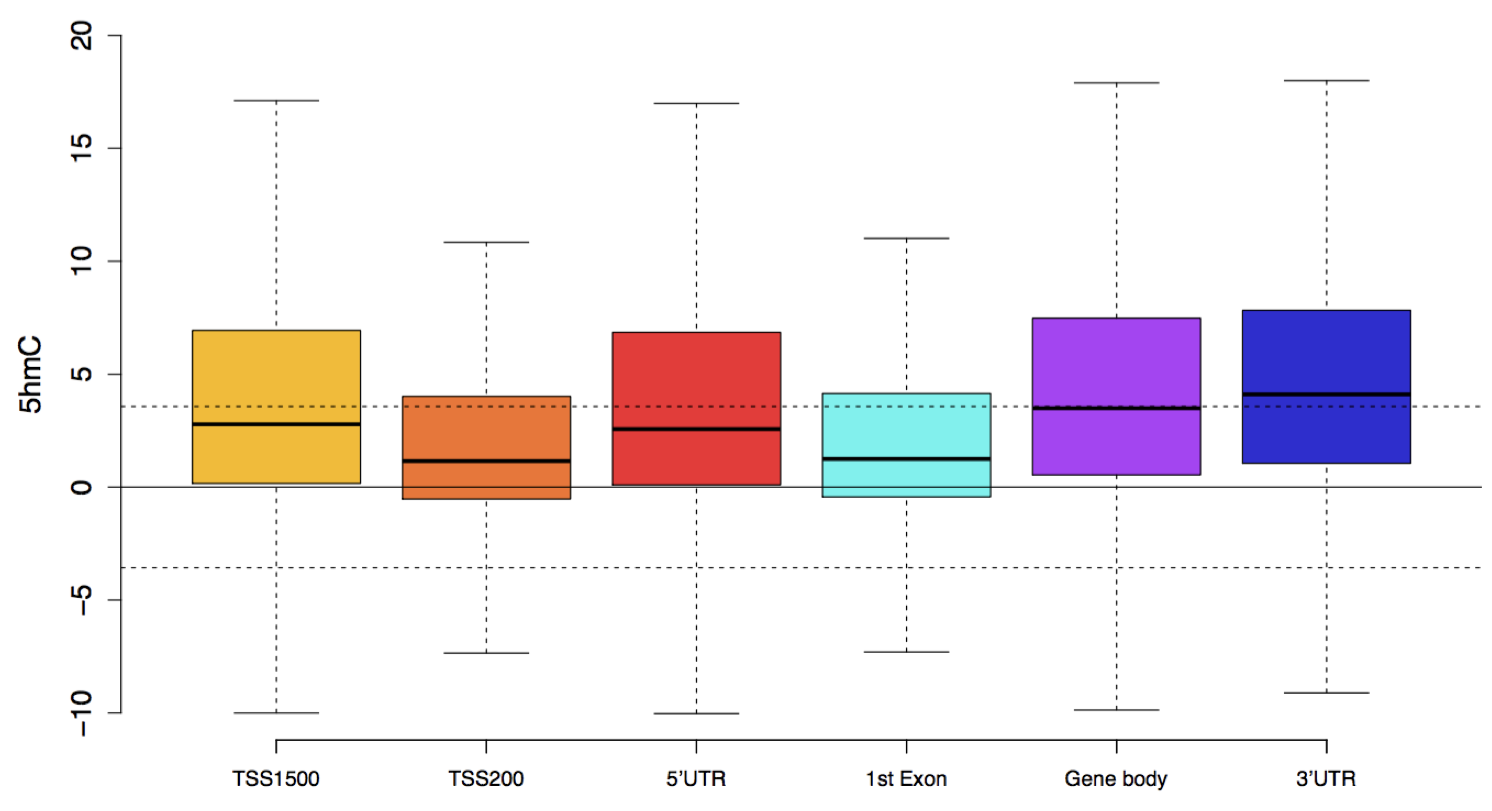


Figure S2: Sites previously characterized as dDMPs in our previous analysis of sodium bisulfite (BS) treated DNA from fetal brain samples (Spiers et al. 2015) show similar trajectories in the current dataset. The four top-ranked (A) hypermethylated dDMPs and (C) hypomethylated dDMPs from our previous analysis (Spiers *et al*., 2015) are shown alongside the data from BS-converted DNA for these sites generated in the current study (B, D). Females are shown in pink, males in blue.

Figure S3: Validation of the DNA modification changes associated with brain development identified in our previous analysis. Regression coefficients for DPC-associated DNA modification changes obtained from Illumina 450K analysis of BS-treated DNA (DNA methylation and DNA hydroxymethylation) are highly between data generated in our previous study (Spiers *et al*., 2015) and the current study. Shown is data for the 28,330 probes previously identified as being significantly associated with DPC in Spiers *et al*., (r = 0.99; *P*-value < 1.00E-200).


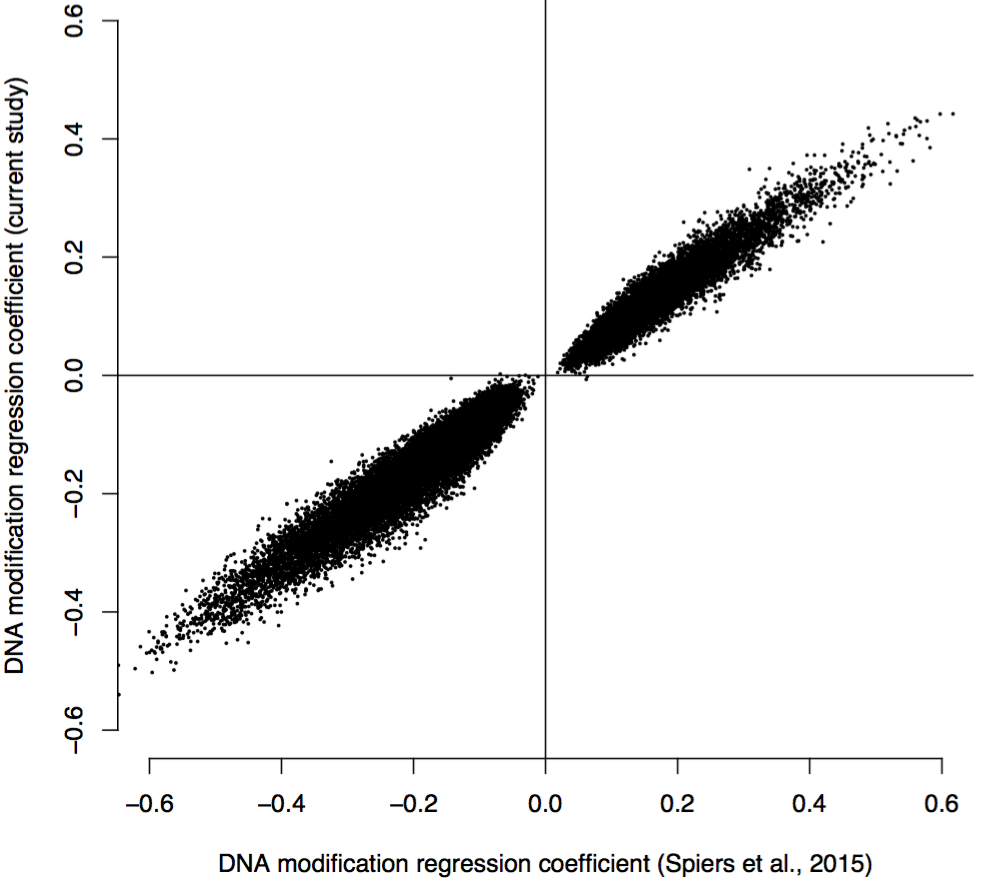


Figure S4: DNA modification and DNA methylation changes associated with brain development are highly correlated. Regression coefficients for DNA modifications and DNA methylation changes associated with brain development are highly correlated for the n = 28,330 probes previously identified as being dDMPs in Spiers *et al*., (r = 0.97; *P*-value- < 1.00E-200).


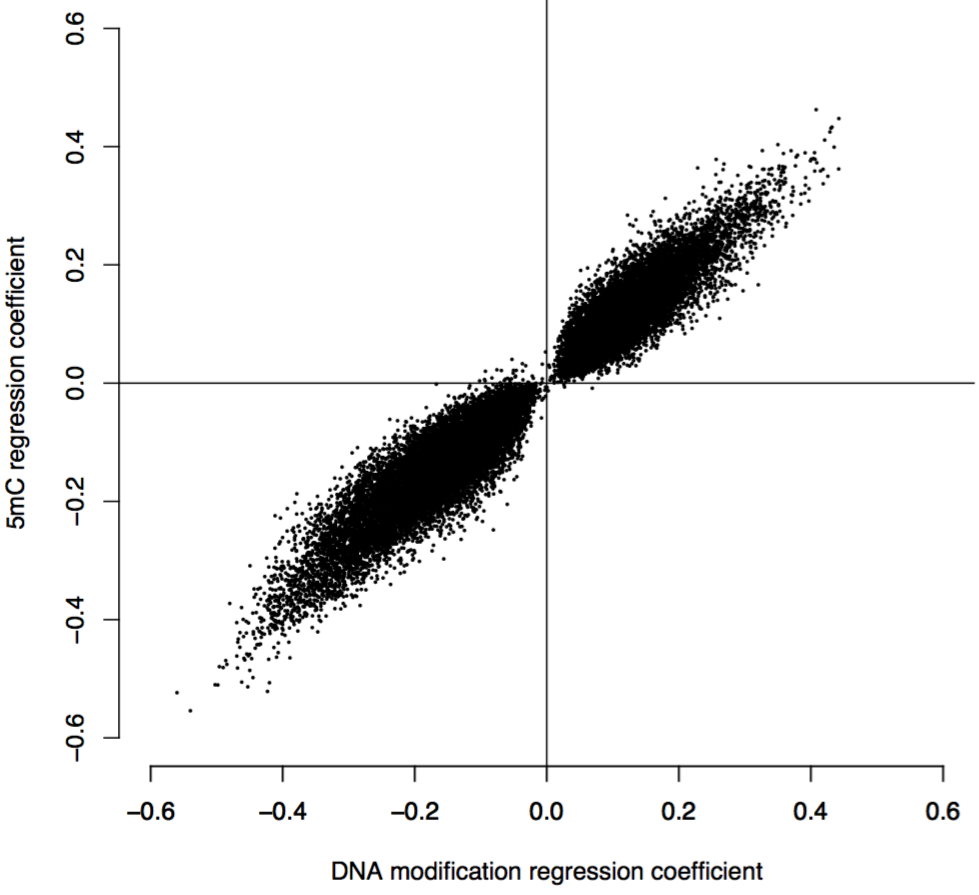


**Figure S5: Changes in DNA hydroxymethylation across brain development is observed at sites previously identified as dDMPs.** For the current study, DPC-association regression coefficients for DNA modifications and DNA hydroxymethylation show a small but significant correlation for the n = 28,255 probes with detectable DNA hydroxymethylation previously defined as significantly associated with DPC in Spiers *et al*., (r = 0.36; *P*-value < 1.00E-200). The contribution of DNA hydroxymethylation to the DNA modification measure can lead to false positive and false negative associations with DPC.


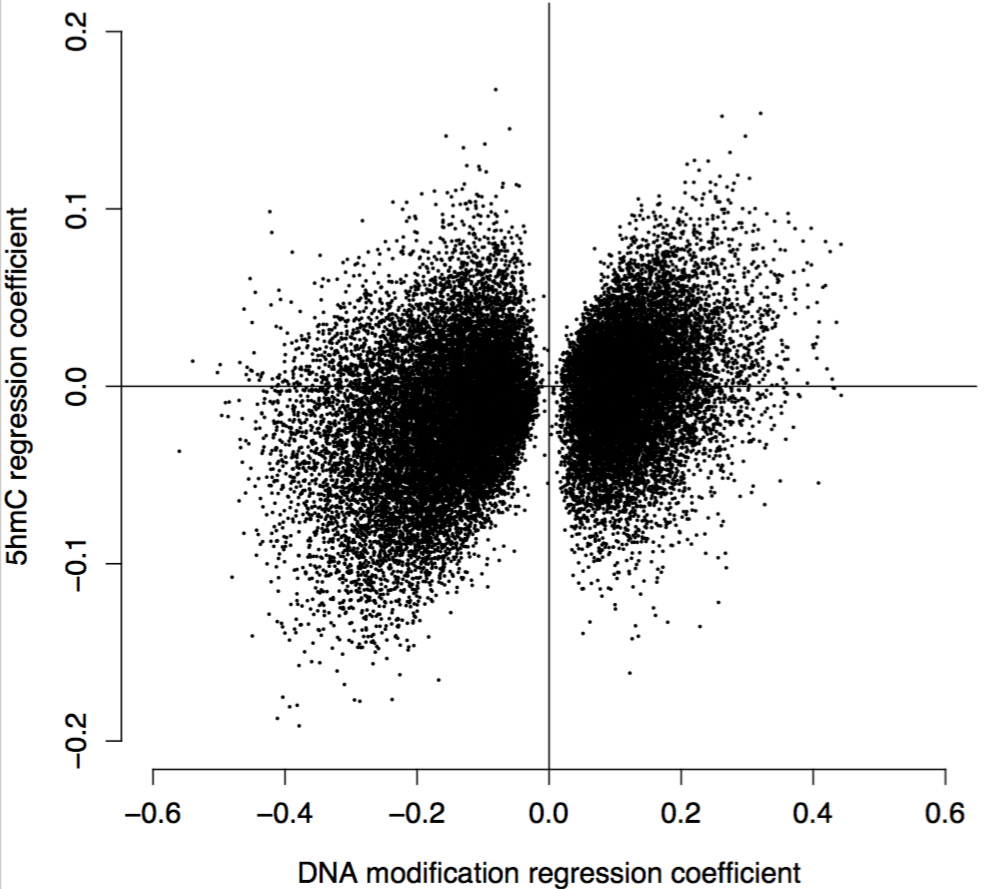


Figure S6: The distribution and direction of fetal brain dDHPs differs across genomic features. A) Compared to the genome average, dDHPs are significantly underrepresented in CpG islands, transcription start-sites and first exons, but significantly enriched in regions not annotated to CpG islands/shores/shelve. B) Overall there is an enrichment of hypohydroxymethylated autosomal dDHPs (*P*-value = 1.29E-08), however CpG island were unique in showing a greater proportion of sites becoming hyperhydroxymethylated with fetal brain development (see also Table S6). (* denotes significance).

**A)**

*
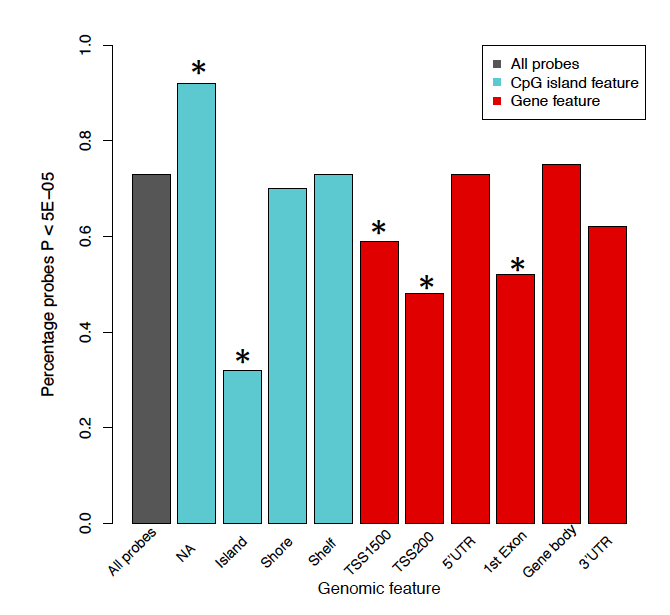
*

**B)**

**
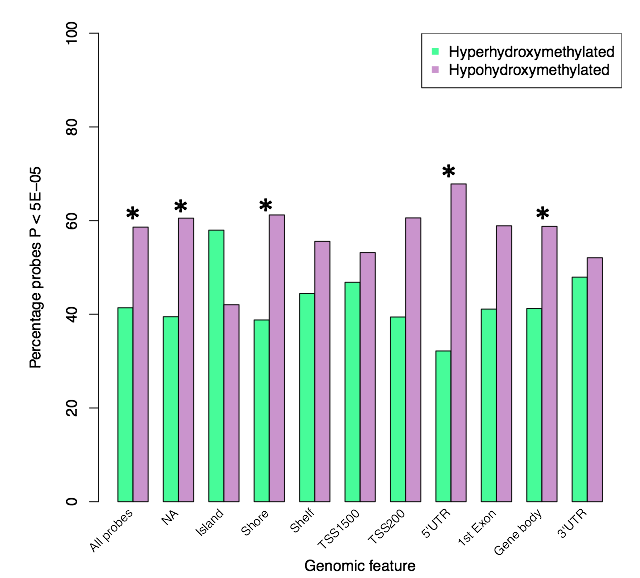
**

Figure S7: The distribution of fetal brain dDHPs across genic features differs as a function of CpG density. The red line represents the average percentage of dDHPs across all autosomal probe sites (0.73%). See also Table S10.

Figure S8: The distribution of fetal brain dDHPs associated with alternative transcription events. The red line represents the average percentage of dDHPs across all autosomal probe sites (0.73%). A3SS = Alternative 3’ splice site, A5SS = Alternative 5’ splice site, AFE = Alternative first exon, ALE = Alternative last exon, CE = Cassette exon, CNE = Constitutive exon, EI = Exon isoforms, II = Intron isoforms, IR = Intron retention, MXE = Mutually exclusive exon. See also Table S12.

Figure S9: Site-specific levels of DNA hydroxymethylation are highly correlated between males and females. Mean DNA hydroxymethylation in male versus female samples is shown for A) all autosomal probes (n =298,972, r = 0.98, *P*-value < 1E-200) and; B) all X-chromosome probes (n = 8,838, r = 0.85, *P*-value < 1E-200).

**A)** **B)**


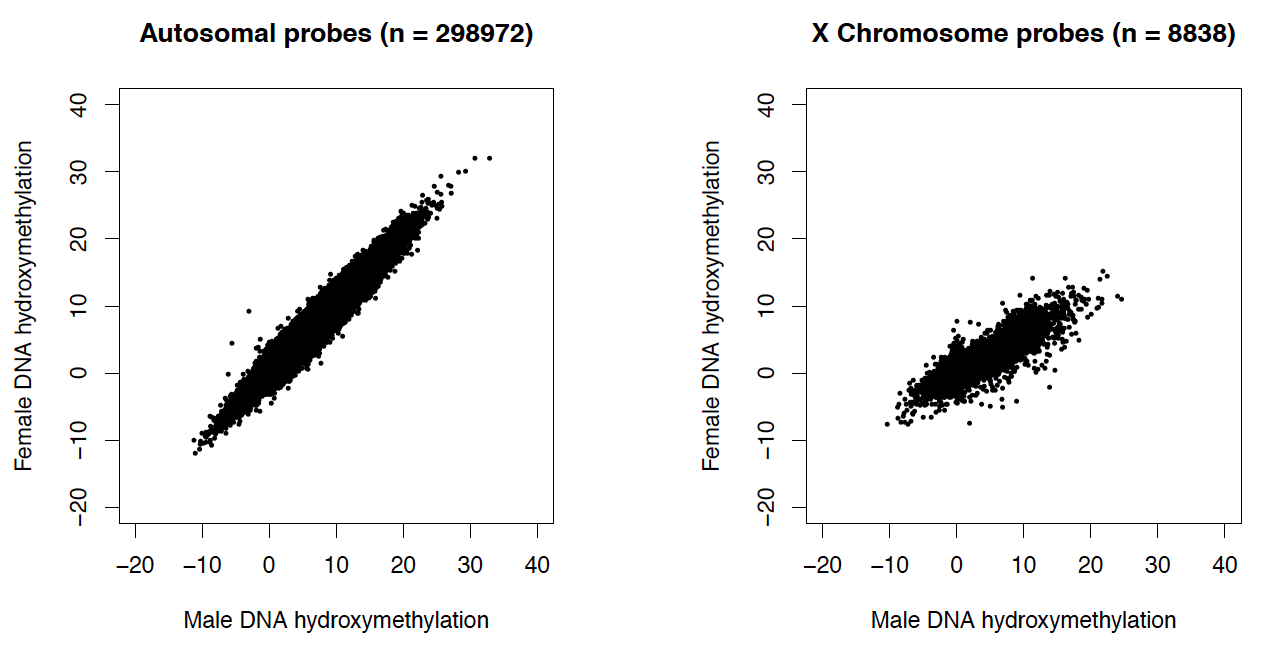


Figure S10: The top-ranked sex-associated DHR spans 720bp (Chr X: 153046175 – 153046895) within the *SRPK3* gene. A full list of sex-associated DHRs is presented in Table S14. The sex-associated DHR is denoted by dashed lines. Chromosomal coordinates correspond to human genome build Feb. 2009 (GRCh37/hg19).

Figure S11: The module eigengene of modules significantly associated with human fetal brain development. See also Fig. 4b.

Figure S12: Module membership is strongly correlated with probe significance


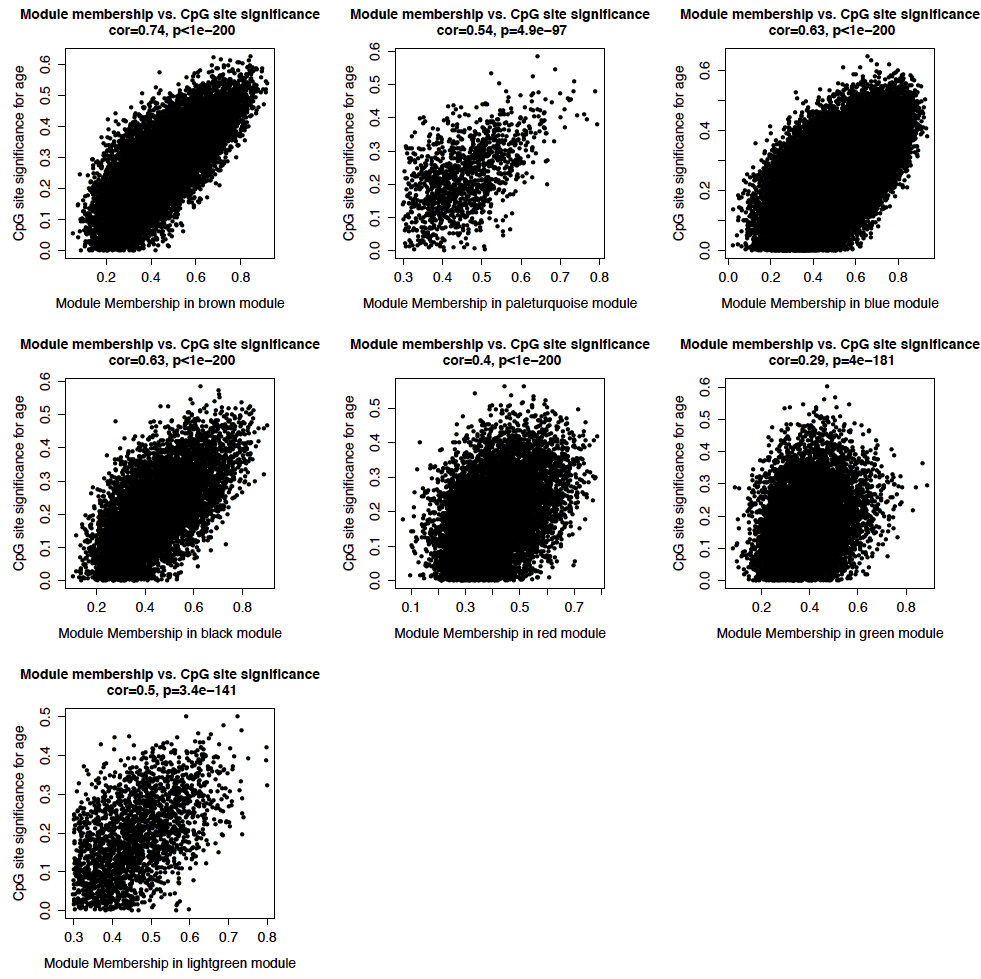


Figure S13: An overview of 5hmC data generation.1. Genomic DNA (1 μg) was purified and denatured, and split into two 500 ng aliquots; 2. Parallel oxBS and BS conversion of the two DNA aliquots was performed using the TrueMethyl® kit (CEGX, Cambridge, UK); 3. DNA conversion efficacy was assessed using a digestion control assay; 4. DNA modifications were then quantified using the Illumina 450K BeadChip. BS and oxBS treated DNA from the same sample were run in the same row on the same array to reduce batch effects; 5. Data quality was assessed through inspecting features including the oxBS and BS beta densities, and genotype correlation between the pretreatments; 6. Poor quality probes and samples were removed prior to; 7. Separate normalization of the oxBS and BS sample data using the “dasen” method of the wateRmelon package. 8. DNA hydroxymethylation at each probe site was calculated by subtraction of the normalized oxBS beta values from the normalized BS beta values, providing a ‘Δ beta’ value corresponding to the level of DNA hydroxymethylation at each probe location. A threshold for defining “detectable” 5hmC was calculated from the 95^th^ percentile the negative beta values; 9. Modifications assessed and clarification of terminology.


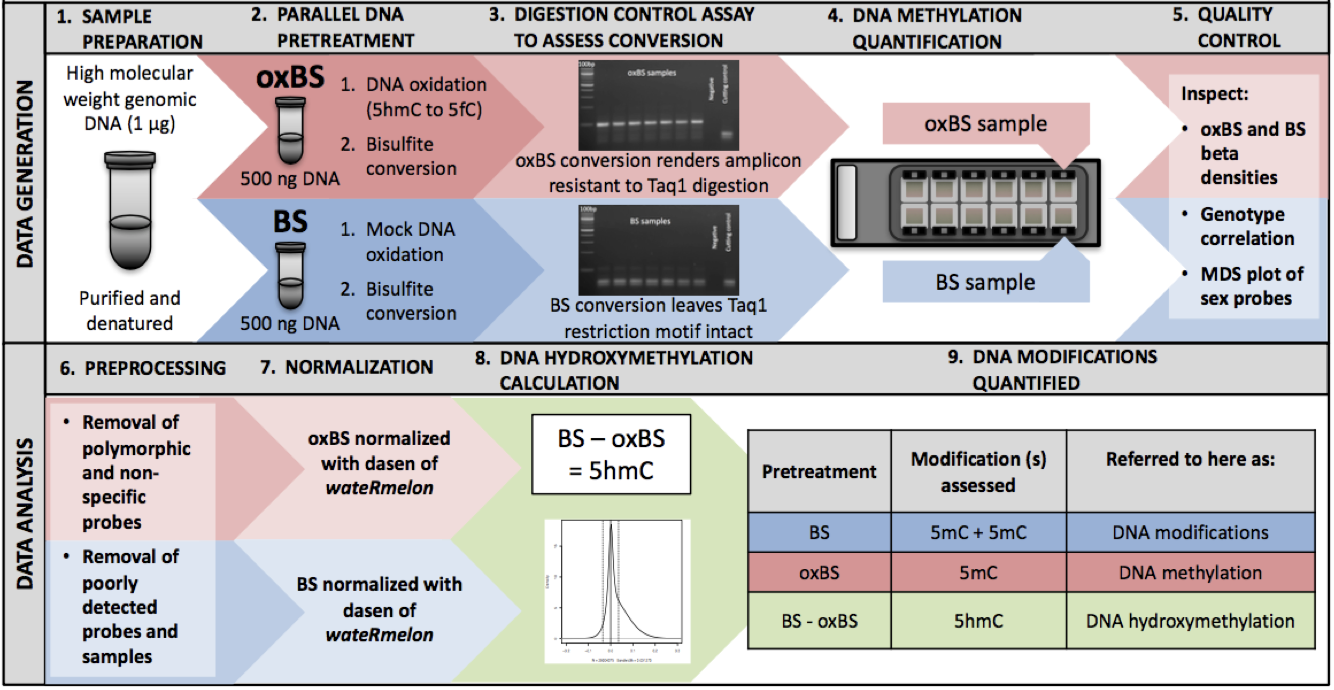


Figure S14: Summary of the fetal brain samples used in this study. Male (n = 35) and female (n = 36) samples were selected for DNA hydroxymethylation quantification. See also Table S19.

Figure S15: Digestion control assay for assessment of oxBS conversion efficacy. A digestion control containing a hydroxymethylated CpG site within a Taq1 restriction motif (5’-TCGA-3’) was included in the cohort subject to oxBS and BS conversion. Following TrueMethyl® treatment, the digestion control was amplified to produce a 100 bp amplicon, which was subjected to digestion with the Taq1 restriction enzyme. For oxBS samples, complete conversion of hydroxymethylated CpG sites to uracil would render the restriction motif resistant to Taq1 digestion, therefore the presence of two bands (40 bp and 60 bp) following digestion would indicate oxidation reaction failure or incomplete conversion. BS treated samples would be expected to be digested because the hydroxymethylated CpG site would not be converted to uracil, leaving the recognition site intact. A) The presence of a single band indicated successful oxidation and complete conversion of template; B) whereas bisulfite treated samples were digested as expected. Ladder = 100 bp.

**A) B)**


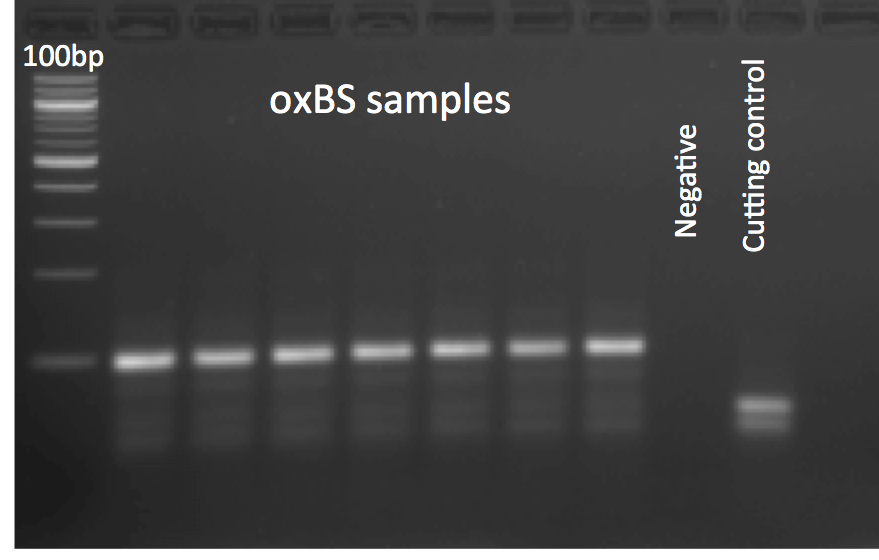

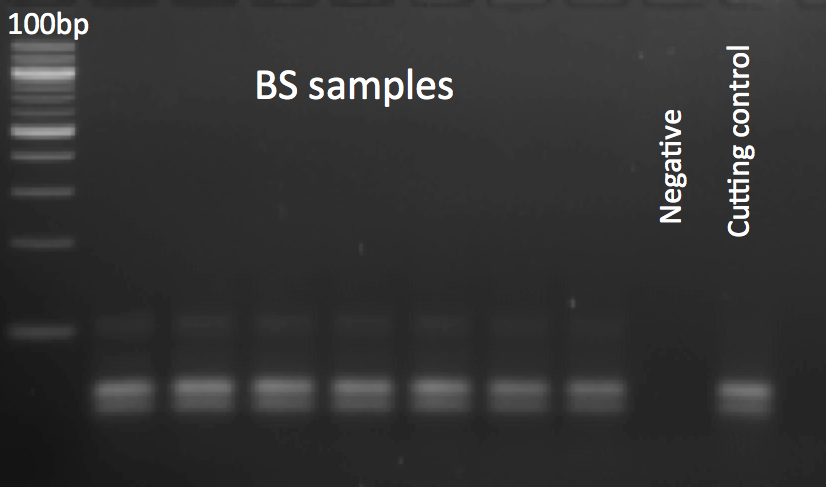


Figure S16: Normalized beta-density plot for DNA modification and DNA methylation data. As expected, a clear shift to the right is observed for DNA modification data, which includes signal from both DNA methylation and DNA hydroxymethylation (probes passing QC are plotted; n = 411,325).

Figure S17: Density plot of DNA hydroxymethylation beta values across the samples profiled in this study. DNA hydroxymethylation was calculated for each sample (Figure S14). A detection confidence threshold based on the lowest fifth percentile in the negative value (DNA hydroxymethylation beta = 0.036) was used to define “detectable” DNA hydroxymethylation (dashed vertical line). A) Density plot for all n = 411,325 probes passing quality control; B) Density plot for n = 307,824 probes remaining following removal of sites displaying no detectable DNA hydroxymethylation in any sample.

**A)**

**B)**
